# New insights into the mechanisms involved in B-type natriuretic peptide elevation and its prognostic value in septic patients

**DOI:** 10.1186/cc13864

**Published:** 2014-05-09

**Authors:** John Papanikolaou, Demosthenes Makris, Maria Mpaka, Eleni Palli, Paris Zygoulis, Epaminondas Zakynthinos

**Affiliations:** 1 Department of Critical Care, School of Medicine, University of Thessaly, University Hospital of Larissa, Biopolis, 41110 Larissa, Thessaly, Greece

## Abstract

**Introduction:**

Elevated plasma B-type natriuretic peptide (BNP) levels in patients with critical sepsis (severe sepsis and septic shock) may indicate septic cardiomyopathy. However, multiple heterogeneous conditions may also be involved in increased BNP level. In addition, the prognostic value of BNP in sepsis remains debatable. In this study, we sought to discover potential independent determinants of BNP elevation in critical sepsis. The prognostic value of BNP was also evaluated.

**Methods:**

In this observational study, we enrolled mechanically ventilated, critically septic patients requiring hemodynamic monitoring through a pulmonary artery catheter. All clinical, laboratory and survival data were prospectively collected. Plasma BNP concentrations were measured daily for five consecutive days. Septic cardiomyopathy was assessed on day 1 on the basis of left and right ventricular ejection fractions (EF) derived from echocardiography and thermodilution, respectively. Mortality was recorded at day 28.

**Results:**

A total of 42 patients with severe sepsis (*N* = 12) and septic shock (*N* = 30) were ultimately enrolled. Daily BNP levels were significantly elevated in septic shock patients compared with those with severe sepsis (*P* ≤0.002). Critical illness severity (assessed by Acute Physiology and Chronic Health Evaluation II and maximum Sequential Organ Failure Assessment scores), and peak noradrenaline dose on day 1 were independent determinants of BNP elevation (*P* <0.05). Biventricular EFs were inversely correlated with longitudinal BNP measurements (*P* <0.05), but not independently. Pulmonary capillary wedge pressures (PCWP) and volume expansion showed no correlation with BNP. In septic shock, increased central venous pressure (CVP) and CVP/PCWP ratio were independently associated with early BNP values (*P* <0.05).

Twenty-eight-day mortality was 47.6% (20 of 42 patients). Daily BNP values poorly predicted outcome; BNP on day 1 > 800 pg/ml (the best cutoff point) fairly predicted mortality, with a sensitivity%, specificity% and area under the curve values of 65, 64 and 0.70, respectively (95% confidence interval = 0.54 to 0.86; *P* = 0.03). Plasma BNP levels declined faster in survivors than in nonsurvivors in both critical sepsis and septic shock (*P* ≤0.002). In septic shock, a BNP/CVP ratio >126 pg/mmHg/ml on day 2 and inability to reduce BNP <500 pg/ml implied increased mortality (*P* ≤0.036).

**Conclusions:**

The severity of critical illness, rather than septic cardiomyopathy, is probably the major determinant of BNP elevation in patients with critical sepsis. Daily BNP values are of limited prognostic value in predicting 28-day mortality; however, fast BNP decline over time and a decrease in BNP <500 pg/ml may imply a favorable outcome.

## Introduction

B-type natriuretic peptide (BNP) is a cardiac hormone with diuretic, natriuretic and vasorelaxing properties. It is considered to be produced by ventricular myocardium in response to increased wall stretch and plays a fundamental role in regulating cardiac filling pressure and intravascular volume homeostasis [1-3]. Therefore, BNP is used widely in cardiology as a valuable biomarker of left ventricular (LV) dysfunction and increased LV filling pressure [2,4-6]. BNP levels may remain high despite appropriate therapy in heart failure, however, suggesting that stimuli other than LV pressure and/or volume overload may be implicated in the release of the peptide [7].

Plasma BNP concentrations may also be considerably high in patients with critical sepsis (henceforth, the term *critical sepsis* is used to include both severe sepsis and septic shock) [1,8]. In such patients, BNP has been proposed as a valuable screening tool to detect underlying cardiac dysfunction (otherwise known as *septic cardiomyopathy*) [9-11]. Several heterogeneous conditions may also account for increased BNP levels, such as the intensity of inflammation *per se*, vasopressors used, renal failure and right ventricular (RV) overload [1,8,12-19]. Therefore, the primary conditions predisposing patients to BNP elevation in sepsis, as well as the associations between them, are largely undetermined. In addition, the diagnostic performance of BNP in predicting sepsis outcomes remains questionable [9-11,19-21].

In this observational study, we sought potential independent determinants of BNP elevation in critical sepsis. We prospectively evaluated the influence of several clinical parameters, which have previously been associated with BNP rise [8-19], on 5-day longitudinal BNP measurements in critical sepsis patients. These predefined parameters included (1) LV and RV systolic function; (2) LV and RV filling pressures and pulmonary artery catheter (PAC)–derived hemodynamic parameters; (3) sepsis severity classification systems, which reflect the intensity of systemic inflammation; (4) renal failure; (5) acute lung injury and acute respiratory distress syndrome; and (5) iatrogenic interventions (fluid and vasopressor infusion and ventilator settings). In order to provide further insight into the influence of noradrenaline infusion on BNP rise in critical sepsis, we also studied BNP levels in patients with hemorrhagic shock requiring noradrenaline support and PAC monitoring. Finally, we investigated the diagnostic role of BNP in predicting mortality in critical sepsis patients.

## Methods

In this single-center observational study, we prospectively examined critically ill patients with severe sepsis and septic shock admitted to our general ICU during a 3-year period from February 2009 to January 2012. The inclusion criteria were (1) requirement of support with mechanical ventilation and (2) need for hemodynamic monitoring by using a PAC (with both criteria met for at least 3 days). Exclusion criteria were (1) younger than 18 years of age; (2) pregnancy; (3) chronic heart disease (coronary artery disease, cardiac failure, severe valvulopathy and/or cardiomyopathy); (4) chronic renal failure; (5) known pulmonary hypertension; (6) diseases of the central nervous system (CNS; for example, meningitis, brain abscess, cerebral hemorrhage), in which BNP levels are difficult to interpret [18]; (7) poor echocardiographic scan quality; and (8) infusion of inotropic agents (dopamine, dobutamine or levosimendan). Patients receiving noradrenaline were included in the study.

Preliminary data in three ICU patients with acute blood loss requiring noradrenaline infusion showed that BNP concentrations in hemorrhagic shock are extremely lower than in septic shock patients. Noradrenaline infusion is considered a potential stimulus for BNP increase [15]; however, its influence on BNP rise in critical sepsis is undefined. Thus, in order to provide further insight into the mechanisms involved in BNP production in critical sepsis, we decided to study BNP kinetics prospectively in a randomization arm of patients with hemorrhagic shock.

Patients with critical sepsis were managed according to the standard protocol of care for sepsis [22], including antibiotics, vasopressors, respiratory support and surgical intervention if indicated. Clinical management decisions were made by the attending physicians, including the intention to place a PAC for diagnosis or monitoring as part of standard care. All cases were discussed daily in a multidisciplinary meeting. Deaths that occurred within 28 days after ICU admission were recorded (that is, 28-day mortality). Patients were considered “survivors” if they survived for at least 28 days after admission.

The study protocol was approved by the Ethical and Educational Committee of the University Hospital of Larissa and conforms to the ethical guidelines of the 1975 Declaration of Helsinki. Patients’ next of kin provided informed consent for all participants.

### Clinical assessment

The patients’ clinical information that we gathered at baseline included age, sex, reason for admission, mean arterial blood pressure (mABP, derived from a radial or femoral artery catheter), heart rate (HR) and arterial blood gas analysis (pH, partial pressure of oxygen (PaO_2_) and partial pressure of carbon dioxide (PaCO_2_)). Respiratory parameters, such as positive end-expiratory pressure (PEEP) and PaO_2_/FiO_2_ ratio (ratio of partial pressure of oxygen in blood to the oxygen concentration during mechanical ventilation, as a marker of oxygenation), were also available at baseline. The Acute Physiology and Chronic Health Evaluation II (APACHE II) score was used as a clinical marker of the severity of disease at the time of admission. Peak noradrenaline dose, fluid balance and renal failure (defined as creatinine level ≥2 mg/dl or requirement for continuous renal substitution therapy) were also recorded at least on day 1. The degree of organ dysfunction or organ failure was quantified by the total maximum Sequential Organ Failure Assessment (SOFA) score [23], defined as the aggregate score of the maximum organ failure scores calculated for each of the six components of the SOFA system during the first 5 days of the study.

### Pulmonary artery catheterization: hemodynamic measurements

The PAC catheter (model 93A-431H-7.5F; Baxter Edwards, Santa Ana, CA, USA), which was equipped with a rapid response thermistor (response time = 50 ms) for the calculation of right ventricular ejection fraction (RVEF), was inserted into the jugular or subclavian vein and remained in place for at least 3 consecutive days. The PAC provided baseline hemodynamic measurements (day 1) from day 1 to day 3, including mean pulmonary arterial pressure (mPAP), cardiac index (CI), Systemic Vascular Resistance Index (SVRI) and pulmonary vascular resistance index (PVRI), as well as longitudinal assessment of RV and LV filling pressures (central venous pressure (CVP) and pulmonary capillary wedge pressure (PCWP), respectively).

Pressure and flow transducers were carefully calibrated before starting each measurement, as previously described [24]. Patients were studied while supine, and zero pressure was measured at atmospheric pressure at the midaxillary line. All hemodynamic measurements were taken at end expiration. Criteria for adequate PCWP measurements were an end-expiratory PCWP less than the end-expiratory diastolic PAP and a similar increase in both PCWP and diastolic PAP during inspiration, validating that the occluded pulmonary artery catheter tip did not reflect zone 1 or zone 2 conditions. The CVP/PCWP ratio, a hemodynamic index of RV dysfunction [25], was also extracted on a daily basis (from day 1 to day 3).

### Assessment of left ventricular and right ventricular systolic function

LV systolic function was assessed by transthoracic echocardiography (Vivid 3 transducer (1.5 to 3.6 MHz); GE Medical Systems, Milwaukee, WI, USA) within 24 hours after the induction of critical sepsis. Studies were analyzed offline by a cardiologist (JP) blinded to patient identity. LV ejection fraction (LVEF) was calculated from the apical four-chamber view by using Simpson’s method of disks and according to recommendations of the American Society of Echocardiography [26]. RVEF was obtained by thermodilution (RVEF I computer; Baxter Edwards) [27] at the same time point as LVEF.

According to a previous classification of LV systolic function in critical sepsis [28], LVEF was defined as normal or slightly reduced (LVEF ≥50%), moderately reduced (LVEF between <50% and ≥35%) and severely reduced (LVEF <35%). In our mechanically ventilated patients, PAC-derived RVEF was graded as normal (≥40%), moderately depressed (≥30% to 39%) or severely depressed (<30%) as previously described [29].

### Radioimmunoassay for B-type natriuretic peptide measurements

Plasma BNP concentration was measured on a daily basis from day 1 to day 5 using a Biosite Triage immunoassay (Biosite Diagnostics, San Diego, CA, USA). During the first 3 days of the study, blood samples were taken at the time when hemodynamic measurements were performed.

### Statistical analysis

The results are expressed as means ± standard error (SE) unless otherwise stated. The Kolmogorov-Smirnov test was used for normality assessment. As appropriate, a χ^2^ test or Fisher’s exact test was used to compare categorical variables, and a *t*-test or Mann-Whitney *U* test was used to compare continuous variables. One-way analysis of variance was used for multiple comparisons. Linear regression analyses were used to determine associations among continuous variables. Multivariate linear regression analysis was used to examine the effect of several univariate predictors in determining BNP measurements independently. Receiver operating characteristic (ROC) curve analysis was performed to evaluate the diagnostic performance of BNP or BNP/CVP ratio in predicting mortality. To evaluate 5-day BNP kinetics among subgroups, mean regression lines were created and compared by using linear mixed model analysis. Univariate and multivariate (backward stepwise selection method with probability for the removal of 0.10) logistic regression analyses were used to determine the association of variables with 28-day mortality. Kaplan-Meier logrank and univariate and multivariate (backward stepwise selection method with probability for removal of 0.10) Cox proportional hazards regression models were used to identify the strongest predictors of overall time-tagged mortality using time to death as a continuous variable. Only the variables with statistically significant associations with mortality in univariate analysis were included in the multivariate models. The statistical software package SPSS 17.0 (SPSS, Chicago, IL, USA) was used.

## Results

Forty-two patients with severe sepsis (*N* = 12) and septic shock (*N* = 30) fulfilled the eligibility criteria and were enrolled in the study. Eleven patients with hemorrhagic shock were also examined. Differences between groups according to their baseline clinical characteristics, admitting etiology and outcome are given in Table 1. Additional file 1 shows the clinical characteristics of our 42 patients in more detail.

**Table 1:** **Clinical characteristics and 28**-**day mortality in septic shock patients (*N*** **= 30), severe sepsis patients (*N*** **= 12) and hemorrhagic shock patients (*N*** **= 11)**^**a**^

**Characteristics**	**Septic shock (*N*** **= 30)**	**Severe sepsis (*N*** **= 12)**	**Hemorrhagic shock (*N*** **= 11)**	***P-*** **value (ANOVA)**
Age, yr	60.9 ± 1.8	58.8 ± 3	40.2 ± 3.7	<0.001^b^
Sex (M/F)	18 (60)/12 (40)	8 (66.7)/4 (33.3)	7 (63.6)/4 (36.4)	0.921
Admitting diagnosis				
Medical critical state	21 (70)	8 (66.7)	0 (0)	
Surgical critical state	7 (23.3)	3 (25)	1 (9.1)	<0.001^b,c^
Multiple trauma	2 (6.7)	1 (8.3)	10 (90.9)	
Etiology of sepsis				
Pneumonia	8 (26.6)	8 (66.7)	–	
Bacteremia/CRS	12 (40)	2 (16.6)	–	
Peritonitis	4 (13.3)	1 (8.3)	–	
Cholecystitis	2 (6.6)	0	–	
Pyelonephritis	1 (3.3)	0	–	
Cellulitis	1 (3.3)	0	–	
Unknown	2 (6.6)	1 (8.3)	–	
Comorbidities in septic patients				
Diabetes	6 (20)	3 (25)	–	
Hepatic disease	4 (13.3)	1 (8.3)	–	
Respiratory disease	7 (23.3)	2 (16.6)	–	
Cancer	4 (13.3)	2 (16.6)	–	
Autoimmune disease	5 (16.6)	1 (8.3)	–	
Glucocorticoid therapy	6 (20)	2 (16.6)	–	
Baseline clinical parameters				
APACHE II score	19.9 ± 0.8	14.8 ± 0.6	9.8 ± 0.7	<0.001^b,c,d^
Peak noradrenaline dose, μg/min	19.7 ± 2.1	–	17.8 ± 2.5	0.619^e^
Fluid balance, ml	5,305.3 ± 218.7	2,802.5 ± 269.9	3,703.6 ± 398.3	<0.001^b,d^
Renal failure	7 (23.3)	4 (33.3)	0 (0)	0.13
Baseline ventilatory parameters				
pH	7.4 ± 0.01	7.39 ± 0.01	7.41 ± 0.01	0.552
PEEP, mmHg	5.93 ± 0.13	5.92 ± 0.23	4.82 ± 0.12	<0.001^b,c^
PaO_2_/FiO_2_ ratio	355.9 ± 13.5	341.5 ± 18.1	368.3 ± 27.9	0.699
Total maximum SOFA score	10.8 ± 0.4	8.9 ± 0.7	8 ± 0.5	0.002^b^
Mortality				
28-day mortality	17 (56.67)	3(25)	3 (27.27)	0.085

^a^ANOVA, Analysis of variance; APACHE II, Acute Physiology and Chronic Health Evaluation II; CRS, Catheter-related sepsis; FiO_2_, Fraction of inspired oxygen; PaO_2_, Partial pressure of oxygen; PEEP, Positive end-expiratory pressure; Renal failure, Creatinine level ≥2 mg/dl or requirement for continuous renal substitution therapy; SOFA, Sequential Organ Failure Assessment. ^b^P < 0.05 (septic shock vs. hemorrhagic shock), ^c^P < 0.05 (severe sepsis vs. hemorrhagic shock) and ^d^P < 0.05 (septic shock vs. severe sepsis), all by one-way ANOVA with Bonferroni *post hoc* test. ^e^*t*-test or χ^2^ test. Continuous data are presented as means ± SE, and categorical data are presented as *n* (%).

PAC-derived hemodynamic measurements on day 1, 3-day recordings of PCWP and CVP and the incidence of LV and RV systolic dysfunction at baseline are listed in Table 2 (for further details see Additional file 1, “The incidence of LV and RV septic cardiomyopathy” section).

**Table 2:** **Echocardiographic, hemodynamic data and serial B**-**type natriuretic peptide measurements in septic shock patients (*N*** **= 30), severe sepsis patients (*N*** **= 12) and hemorrhagic shock patients (*N*** **= 11)**^**a**^

	**Septic shock (*N*** **= 30)**	**Severe sepsis (*N*** **= 12)**	**Hemorrhagic shock (*N*** **= 11)**	***P-*** **value (one-way ANOVA)**
Ventricular systolic function on day 1				
LVEF, %	63.97 ± 2.28	60 ± 2.46	72.45 ± 1.67	0.021^b^
LVEF ≥50%	26 (86.67)	11 (91.67)	10 (90.91)	0.439
LVEF ≥35% to 49%	3 (10)	1 (8.33)	1 (9.09)	
LVEF <35%	1 (3.33)	0	0	
RVEF, %	32.83 ± 6.52	36.25 ± 1.26	41.73 ± 1.81	<0.001^c^
RVEF ≥40%	7 (23.33)	2 (16.67)	8 (72.73)	0.004^c^
RVEF ≥30% to 39%	13 (43.33)	9 (75)	3 (27.27)	
RVEF <30%	10 (33.33)	1 (8.33)	0	
Baseline hemodynamic measurements				
mABP, mmHg	69.5 ± 1.4	74.17 ± 3	75 ± 1.13	0.072
mPAP, mmHg	24.3 ± 0.73	23.33 ± 0.88	19.27 ± 1.48	0.004^c^
SVI, ml/m^2^	39.12 ± 1.52	44.44 ± 2.06	31.14 ± 1.18	<0.001^b,c^
CI, L/min/m^2^	4.56 ± 0.16	4.37 ± 0.17	3.44 ± 0.13	<0.001^b,c^
SVRI, dyn/s/cm^5^/m^2^	1,078.2 ± 46.1	1,183.1 ± 60.4	1,580.2 ± 64.3	<0.001^b,c^
PVRI, dyn/s/cm^5^/m^2^	221.38 ± 17.3	185.3 ± 24.1	229.03 ± 31.97	0.466
LVSWI, g/m/m^2^	30.6 ± 1.4	37.1 ± 2.3	27.7 ± 1.1	0.013^b,d^
Serial PCWP and CVP measurements, mmHg				
PCWP (day 1)	12 ± 0.62	13.5 ± 0.64	9.55 ± 0.92	0.013^b^
PCWP (day 2)	13.8 ± 0.53	13.92 ± 0.54	11.36 ± 0.73	0.027^c^
PCWP (day 3)	13.5 ± 0.50	14.08 ± 0.48	12.3 ± 0.86	0.222
CVP (day 1)	9.67 ± 0.54	10.25 ± 0.43	7.82 ± 0.58	0.056
CVP (day 2)	9.64 ± 0.42	10.17 ± 0.30	9.35 ± 0.56	0.13
CVP (day 3)	10.73 ± 0.6	10.95 ± 0.57	9.64 ± 0.69	0.464
Serial BNP measurements, pg/ml				
BNP (day 1)	1,145.57 ± 101.43	311.33 ± 41.57	56.82 ± 17	0.001^c,d^
BNP (day 2)	1,232.6 ± 142.37	320.67 ± 39.02	56.4 ± 12.17	0.001^b,c,d^
BNP (day 3)	1,062.52 ± 136.68	290.75 ± 42.84	74.8 ± 21.1	0.001^c,d^
BNP (day 4)	944.77 ± 156.01	288.92 ± 54.88	42 ± 13.58	0.002^c,d^
BNP (day 5)	778.84 ± 145.82	257 ± 64.66	34.17 ± 10.76	0.002^c,d^

^a^ANOVA, Analysis of variance; BNP, B-natriuretic peptide; CI, Cardiac index; CVP, Central venous pressure; LVEF, Left ventricular ejection fraction; LVSWI, Left Ventricular Stroke Work Index; mABP, Mean arterial blood pressure; mPAP, Mean pulmonary artery pressure; PCWP, Pulmonary capillary wedge pressure; PVRI, Pulmonary Vascular Resistance Index; RVEF, Right ventricular ejection fraction; SVI, Stroke volume index; SVRI, Systemic vascular resistance index. ^b^*P* < 0.05, severe sepsis vs. hemorrhagic shock; ^c^*P* < 0.05, septic shock vs. hemorrhagic shock; and ^d^*P* < 0.05, septic shock vs. severe sepsis (all with Bonferroni *post hoc* analysis). Continuous data are presented as means ± SE, and categorical data are expressed as *n* (%).

### Serial B-type natriuretic peptide measurements and kinetics in septic shock, severe sepsis and hemorrhagic shock patients

**Figure 1:**
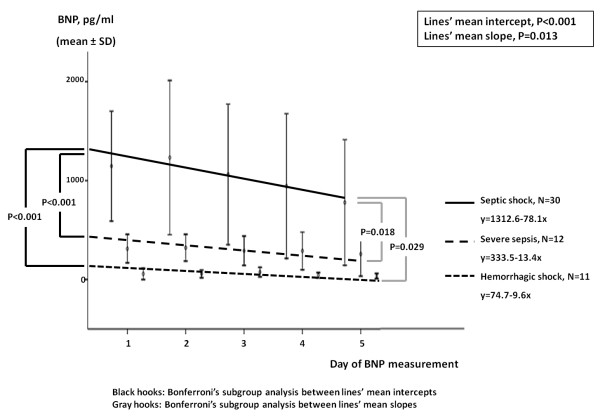
**Five-day B-type natriuretic peptide kinetics in patients with septic shock (*N*** **= 30), severe sepsis (*N*** **= 12) and hemorrhagic shock (*N*** **= 11).** Circles and vertical lines indicate mean B-type natriuretic peptide (BNP) values and standard deviations (SD), respectively. BNP kinetics are indicated by the corresponding mean regression lines for septic shock (solid line), severe sepsis (dashed line) and hemorrhagic shock (dotted line). Septic shock’s mean regression line represents greater mean intercept and steeper mean slope than severe sepsis and hemorrhagic shock regression lines (1,312.6 vs. 333.5 pg/ml and 74.7 pg/ml, respectively, *P* < 0.001; and −78.1 pg/ml/day vs. −13.4 and −9.6 pg/ml/day, respectively, P ≤ 0.029). Intercept of the regression line, BNP value where the regression line crosses the *y*-axis at theoretical day 0; Slope of the regression line, Rate at which BNP values change day after day. Black hooks: Bonferroni's subgroup analysis between lines' mean intercepts. Gray hooks: Bonferroni's subgroup analysis between lines' mean slopes.

Longitudinal BNP measurements in septic shock patients (*N* = 30), severe sepsis patients (*N* = 12) and hemorrhagic shock patients (*N* = 11) are provided in Table 2. Plasma BNP levels were drastically elevated in septic shock patients compared to either severe sepsis patients or hemorrhagic shock patients on any study day (*P* ≤ 0.002). Five-day BNP kinetics demonstrated a significantly steeper decline over time in septic shock patients than in severe sepsis or hemorrhagic shock patients (Figure 1).

### Clinical determinants of B-type natriuretic peptide in critical sepsis and septic shock

Among several clinical parameters examined in critical sepsis patients overall (*N* = 42), APACHE II score, peak noradrenaline dose, maximum SOFA score [23], LVEF, LV Stroke Work Index (LVSWI) score and RVEF were found to correlate significantly with serial BNP measurements in univariate linear regression analysis (Table 3). LV and RV filling pressures (PCWP and CVP, respectively) were not correlated with their corresponding BNP values (*P* > 0.05). Baseline hemodynamic parameters (mABP, mPAP, CI, SVRI and PVRI), fluid balance, PEEP levels, PaO_2_/FiO_2_ ratio and sepsis-induced renal failure were not associated with BNP either (*P* > 0.05).

**Table 3:** **Clinical determinants of B**-**type natriuretic peptide in critical sepsis (*N*** **= 42)**^**a**^

	**Clinical determinants of BNP**	**Univariate linear regression analysis**			**Multivariate linear regression analysis**		
		***r***	***R***^**2**^	***P-*** **value**	**β**	**Β (95% CI)**	***P*-value**
BNP (day 1)	APACHE II score	0.553	0.284	<0.001	0.066	9 (−15 to 33)	0.455
	SOFA score	0.662	0.438	<0.001	0.176	42.6 (−4 to 90)	0.073
	Noradrenaline dose	0.886	0.784	<0.001	0.731	33.4 (23 to 44)	<0.001
	LVSWI score	0.417	0.174	0.006	−0.003	−0.2 (−12 to 12)	0.968
	RVEF	−0.490	0.240	0.001	−0.02	−1.9 (−20 to 16)	0.829
BNP (day 2)	APACHE II score	0.557	0.310	<0.001	0.154	26.7 (−15 to 69)	0.207
	SOFA score	0.639	0.409	<0.001	0.233	72.5 (−9 to 154)	0.080
	Noradrenaline dose	0.762	0.58	<0.001	0.502	29.4 (11 to 48)	0.003
	LVEF	−0.363	0.132	0.009	−0.138	−9.3 (−28 to −9)	0.312
	LVSWI score	0.389	0.151	0.011	−0.038	−3.4 (−25 to 18)	0.745
	RVEF	−0.486	0.236	0.001	0.031	−3.9 (−34 to 42)	0.835
BNP (day 3)	APACHE II score	0.645	0.416	<0.001	0.262	40.6 (6 to 75)	0.022
	SOFA score	0.708	0.502	<0.001	0.254	68 (0 to 138)	0.05
	Noradrenaline dose	0.775	0.601	<0.001	0.430	23.8 (7 to 41)	0.007
	LVEF	−0.379	0.143	0.009	−0.112	−6.5 (−21 to 8)	0.373
	LVSWI score	0.353	0.125	0.027	−0.002	−0.14 (−17 to 17)	0.987
	RVEF	−0.504	0.254	0.001	−0.031	−3.7 (−35 to 28)	0.815
BNP (day 4)	APACHE II score	0.703	0.494	<0.001	0.263	42.4 (0 to 85)	0.05
	SOFA score	0.678	0.459	<0.001	0.191	47.6 (−20 to 115)	0.161
	Noradrenaline dose	0.801	0.642	<0.001	0.449	24.9 (8 to 42)	0.006
	RVEF	−0.467	0.218	0.003	−0.133	−15.7 (−41 to 10)	0.223
BNP (day 5)	APACHE II score	0.688	0.446	<0.001	0.334	52.9 (7 to 99)	0.025
	SOFA score	0.662	0.438	<0.001	0.255	55.6 (−9 to 120)	0.089
	Noradrenaline dose	0.720	0.518	<0.001	0.295	15.2 (−2 to 32)	0.083
	LVEF	−0.363	0.131	0.022	−0.192	−19.4 (−50 to 11)	0.203
	RVEF	−0.485	0.235	0.003	−0.029	−1.4 (−16 to 13)	0.843

^a^APACHE II, Acute Physiology and Chronic Health Evaluation Score II; B,β, Unstandardized and standardized β coefficients, respectively; BNP, B-natriuretic peptide; CI = Confidence interval; LVEF, Left ventricular ejection fraction; LVSWI, Left Ventricular Stroke Work Index; Noradrenaline dose, Peak noradrenaline dose on day 1; *r* = Pearson’s correlation coefficient; *R*^2^ = Coefficient of determination; RVEF, Right ventricular ejection fraction; SOFA, Sequential Organ Failure Assessment. Clinical parameters associated significantly with serial BNP measurements on univariate analysis and independent determinants of BNP on the corresponding multivariate regression models.

BNP determinants in univariate analysis were included in multivariate linear regression models (one model for each day of BNP measurement) (Table 3). The intensity of critical illness, as indicated by APACHE II and maximum SOFA scores [23], as well as peak noradrenaline dose on day 1, showed independent associations with BNP values. Septic cardiomyopathy, as assessed by LVEF, LVSWI and RVEF, did not exert any independent effect on BNP values.

Five-day BNP kinetics according to grading (severity) scales of peak noradrenaline dose, APACHE II score and RVEF on day 1 and maximum SOFA score are provided in Additional file 2. The relationship between the percentage daily changes (relative to baseline) in SOFA scores (indicating the evolution of organ dysfunction) and BNP levels during the initial 5 days is illustrated in Additional file 3.

When the analysis was restricted in the subset of septic shock patients (*N* = 30), the severity of critical illness continued to influence BNP levels independently (see also Additional file 1, “Clinical determinants of BNP in septic shock” section, and Additional file 4 for further details). Septic cardiomyopathy was a significant but not independent determinant of BNP concentrations. LV filling pressures continued to show no correlation with corresponding BNP values. Interestingly, CVP on day 1 and CVP/PCWP ratio [25] on day 2 were independently associated with corresponding BNP values.

### Mortality in critical sepsis and septic shock

In the present study, 28-day mortality was 47.6% (20 of 42 patients) in critical sepsis patients overall (*N* = 42) and 56.7% (17 of 30 patients) in the subset of septic shock patients (*N* = 30). The comparisons of patient characteristics according to 28-day survival are presented in Additional file 5 (for further details, see Additional file 1, “Determinants of mortality in critical sepsis” section). Critical illness severity (as indicated by APACHE II and SOFA scores), peak noradrenaline dose on day 1, higher BNP levels on day 1 and reduced RVEF were significant univariate determinants of 28-day mortality. Among these univariate predictors, peak noradrenaline dose, BNP and RVEF independently predicted 28-day mortality in a multivariate Cox regression model. Reduced RVEF was the strongest independent predictor in a second Cox multivariate survival analysis (Table 4). Additional file 6 illustrates the Kaplan-Meier 28-day survival curves of the 42 patients with critical sepsis, stratified according to BNP, RVEF and peak noradrenaline dose on day 1.

**Table 4:** **Cox multivariate survival models examining the effect of univariate determinants and independent predictors of 28**-**day mortality in critical sepsis patients (*N*** **= 42)**^**a**^

	**Hazard ratio**	**95% CI**	**Wald statistic**	***P-*** **value**
Univariate clinical determinants of 28-day mortality				
APACHE II score	1.12	0.98 to 1.29	2.75	0.097
Maximum SOFA score	1.13	0.87 to 1.47	0.82	0.366
Peak noradrenaline dose on day 1	1.09	1.01 to 1.17	4.59	0.032
RVEF	0.89	0.8 to 0.99	4.79	0.029
BNP on day 1	0.988	0.996 to 1.000	4.91	0.027
Independent clinical predictors of 28-day mortality				
RVEF	0.873	0.78 to 0.97	6.18	0.013
Peak noradrenaline dose on day 1	1.085	1.01 to 1.17	5.11	0.024
BNP on day 1	0.999	0.997 to 1.000	3.16	0.076

^a^APACHE II, Acute Physiology and Chronic Health Evaluation II; BNP, B-type natriuretic peptide; CI, Confidence interval; RVEF, Right ventricular ejection fraction; SOFA score, Total maximum Sequential Organ Failure Assessment score. Among all univariate determinants, RVEF, BNP and peak noradrenaline dose on day 1 independently predicted 28-day mortality. RVEF was the strongest independent predictor among them.

### Diagnostic performance of B-type natriuretic peptide in predicting mortality in critical sepsis and septic shock

**Figure 2:**
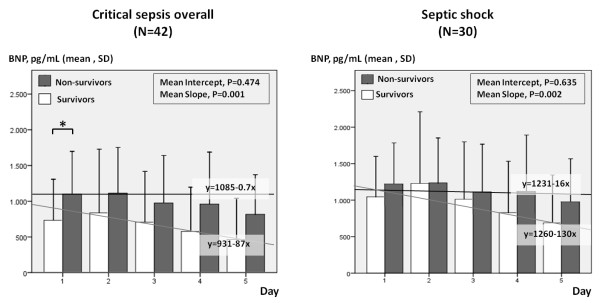
**Daily B-type natriuretic peptide measurements and 5-day B-type natriuretic peptide kinetics in patients with overall critical sepsis (left) and septic shock (right), divided by 28-day mortality.** Bars and vertical lines indicate mean B-type natriuretic peptide (BNP) values and standard deviations (SD), respectively. Significant BNP differences on any study day (*P* < 0.05) are marked with asterisks. Five-day BNP kinetics are indicated by the corresponding mean regression lines (gray in survivors and black in nonsurvivors). Mean regression lines represent similar mean intercepts, yet significantly steeper mean slopes in survivors than in nonsurvivors, either in overall critical sepsis patients (*P* = 0.001) or in septic shock patients (*P* = 0.002). The intercept of the regression line is the BNP value where the regression line crosses the *y*-axis on theoretical day 0. The slope of the regression line is the rate at which BNP values change day after day.

In critical sepsis patients, BNP concentrations on day 1 were significantly higher in 28-day nonsurvivors than in survivors (1,099.5 pg/ml vs. 732.4 pg/ml; *P* = 0.049); however, BNP levels showed no significant differences from day 2 to day 5 (Figure 2, left panel). ROC curve analysis (Additional file 7) showed that BNP values were of limited diagnostic accuracy in predicting 28-day mortality. BNP >800 pg/ml on day 1 (the best cutoff point) and >840 pg/ml on day 2 predicted mortality fairly well (sensitivity (%), specificity (%) and area under the curve (AUC) = 65, 64 and 0.70 (95% confidence interval (CI) = 0.54 to 0.86); *P* = 0.03) and sensitivity (%), specificity (%) and AUC = 65, 64 and 0.68 (95% CI = 0.52 to 0.84); *P* = 0.044) for days 1 and 2, respectively). In the subgroup of septic shock patients, BNP concentrations did not differ between nonsurvivors and survivors on any study day (Figure 2, right panel) and showed no prognostic value in ROC analysis (Additional file 7). Interestingly, 5-day BNP kinetics presented a significantly steeper decline in survivors compared to nonsurvivors in critical sepsis patients overall (*P* = 0.001) (Figure 2, left panel) and in the subset of septic shock patients (*P* = 0.002) (Figure 2, right panel).

In septic shock patients, we introduced two simple clinical markers that showed significant prognostic value for mortality: critical sepsis BNP concentration and BNP/CVP ratio. Critical BNP concentration was defined as the lowest 5-day BNP level in each septic shock patient. Inability to reduce BNP below the critical threshold of 500 pg/ml predicted 28-day mortality with sensitivity (%), specificity (%) and AUC of 82, 62 and 0.74 (95% CI = 0.55 to 0.93; *P* = 0.028). In addition, BNP/CVP >126 pg/ml/mmHg on day 2 predicted mortality with sensitivity (%), specificity (%) and AUC of 73, 77, 0.73 (95% CI = 0.53 to 0.94; *P* = 0.036).

## Discussion

The main findings of our study are as follows. (1) The severity of critical illness is probably the main determinant of BNP rise in critical sepsis patients. Septic cardiomyopathy is associated with BNP rise, although not independently, whereas LV filling pressures do not correlate with the corresponding BNP concentrations. Noradrenalin dose may not be a stimulus of BNP secretion *per se*. (2) Baseline BNP elevation predicts mortality fairly well; yet, 5-day BNP kinetics demonstrate a significantly faster decline over time in survivors than in nonsurvivors. (3) In the subset of septic shock patients, although BNP levels were enormously elevated, they had no prognostic implication. In these patients, an increased BNP/CVP ratio on day 2 might serve as an early prognostic marker for mortality, whereas inability to reduce BNP below the critical threshold of 500 pg/ml may also imply increased mortality.

In the present study, we found BNP concentrations to be considerably elevated in septic shock patients compared with severe sepsis patients on every single day of assessment (*P* ≤ 0.002). This finding differs from the findings of McLean *et al*. [21], who reported no escalation of BNP levels from severe sepsis to septic shock, but it is consistent with the findings in the studies of Pirracchio *et al*. [16] and Ueda *et al*. [30]. As sepsis syndrome evolves and the patient’s condition continues to deteriorate, several mechanisms may be involved in BNP elevation, such as proinflammatory cytokine oversecretion [8,12-14], ensuing systolic and diastolic biventricular dysfunction [9,10,31], altered BNP clearance [16], renal failure [32] and sepsis-associated acute lung injury or acute respiratory distress syndrome [18]. In addition, several medical interventions employed with the aim of treating sepsis [22] may trigger BNP release, such as volume overresuscitation [17,19] and high PEEP levels (both of which result in RV overload [33]), and catecholamine infusion [15].

In our series, the severity of sepsis (as indicated by increased APACHE II and maximum SOFA scores) and high noradrenaline levels on day 1 were independent determinants of serial BNP values. In addition, increased right-side filling pressures (CVP and CVP/PCWP ratio [25]) were independently associated with early BNP elevation in the subset of septic shock patients. Sepsis-induced LV and RV systolic dysfunction, although significantly associated with serial BNP measurements, were not independent predictors of BNP elevation. In contrast to previous data [32], demonstrating a correlation between BNP and PCWP in critically ill non-cardiac patients with preserved renal function, but in line with other previous reports [8,11,34,35], we did not detect any relationship between serial PCWP measurements and the corresponding BNP values.

Our study provides evidence that it is the severity of critical illness rather than cardiac dysfunction that accounts for BNP release in the setting of critical sepsis (Table 3 and Additional file 4). In addition, the results of our study suggest that, in patients with septic shock, RV overload may play a pivotal role in early BNP rise and therefore should be carefully evaluated (Additional file 4). Researchers in previous studies have reported that BNP can be increased [8,12,14,16] and cardiac contractility may be depressed [36] secondarily to the activation of several inflammatory mediators. On this ground, both septic cardiomyopathy and high BNP levels may be epiphenomena in severe sepsis patients. Unfortunately, we did not assess specific inflammatory markers [37] in our study.

Catecholamine oversecretion in critical sepsis patients may adversely influence cardiac function, coagulation (hypercoagulability and thrombus formation), immune system (immunomodulation and stimulation of bacterial growth) and metabolism (increase in cellular energy expenditure, hyperglycemia and impaired glucose tolerance, muscle catabolism, increased lipolysis and hyperlactatemia). Noradrenaline infusion may further impair organ function and cardiovascular homeostasis [38]. In an effort to better comprehend the impact of noradrenaline infusion on BNP production in septic shock patients, we assessed BNP concentrations in 11 patients with hemorrhagic shock. The peak noradrenaline dose was similar between the two types of shock (Table 1); however, BNP levels were extremely higher (approximately 20-fold greater) in septic shock patients than in hemorrhagic shock patients (Table 2 and Figure 1). Our findings suggest that it is neither noradrenaline infusion *per se*, as previously suggested [15], nor the severity of circulatory collapse, but rather the inflammatory nature of shock that primarily accounts for BNP elevation in critical sepsis patients [8,12-14]. Furthermore, in our critically septic patients, the level of vasopressor support was independently associated with mortality in the Cox proportional hazards analysis of survival (Table 4). Therefore, we hypothesize that peak noradrenaline dose on day 1 should be considered as another marker of the intensity of critical sepsis (along with the APACHE II and SOFA clinical grading systems, which were also independent determinants for BNP rise in this study) rather than an index of circulatory failure or an independent upregulating factor in BNP rise.

Ueda and co-workers [30], in a study in which they examined BNP trends in severe sepsis and septic shock patients, found that the BNP levels peaked on day 2 and decreased gradually afterward. Our data are in line with these findings; yet, we report greater BNP values on each study day. In the present study, instead of repeated measures of ANOVA [30], we used linear mixed model analysis to evaluate 5-day BNP kinetics, as the former has been criticized for producing biased results due to missing data (deceased patients) during the study period [39]. We found that BNP kinetics differed significantly in the two subgroups of critical sepsis patients, as the BNP concentrations demonstrated significantly steeper declines in septic shock patients than in severe sepsis patients (Figure 1). These findings may suggest a relatively steady state of BNP kinetics over time in severe sepsis patients, whereas the onset of septic shock is probably characterized by bursts of BNP secretion.

It has been reported previously that plasma BNP concentration on day 2 [10,28,30], on day 3 [9,10] and even on day 5 [9] predicts mortality in septic shock patients. In contrast to these results, McLean *et al*. [21] and Rudiger *et al*. [34] failed to demonstrate any predictive utility of BNP concentration. In a recent meta-analysis [40], Wang *et al*. suggested that elevated BNP during the initial 5 days from the onset of critical sepsis may be a powerful predictor of mortality in septic patients; however, an ideal cutoff BNP value was not determined. Our data provide evidence that daily BNP levels are of limited diagnostic value in predicting 28-day mortality. In critical sepsis patients, early BNP elevation >800 pg/ml (day 1) and >840 pg/ml (day 2) may predict mortality and should be assessed. In the subgroup of septic shock patients, however, BNP level showed no prognostic value in ROC analysis (Additional file 7). Instead, 5-day BNP kinetics demonstrated a significant decline in survivors compared to nonsurvivors in both critical sepsis and septic shock patients (Figure 2). A plausible explanation for this finding is that isolated BNP values are likely to provide “instant images” of the severity of the disease and can be affected by several factors in critical illness. However, persistent elevation of BNP over time may imply intractable critical illness, despite appropriate therapeutic interventions, and, in this respect, it may be of great prognostic value.

In line with the discussion above, we show that the daily BNP percentage changes (relative to baseline values) corresponded well with the evolution of organ dysfunction assessed by SOFA score percentage alterations from baseline (Additional file 3). Notably, the 5-day BNP trends in our septic patients (Figure 2) are comparable to the previously reported APACHE scores in survivors and nonsurvivors [36]. These findings may suggest that the intensity of critical illness is probably the common denominator of BNP rise and decrease in critical sepsis patients. Furthermore, our findings provide evidence that an inability to reduce BNP levels below the critical threshold of 500 pg/ml may indicate poor outcomes in septic shock patients. Accordingly, prolonged BNP elevation should be assessed carefully in critical sepsis patients because it may imply an untreated underlying pathology, despite evidence of clinical improvement.

In the present study, we found that a BNP/CVP ratio >126 pg/ml/mmHg, measured on day 2, demonstrated potential prognostic significance in septic shock patients. CVP monitoring is the cornerstone for fluid resuscitation in septic shock patients [22]; thus the BNP/CVP ratio is easy to calculate. The prognostic importance of the BNP/CVP ratio is that it may provide prognostic information early in the course of septic shock. Although the greater the CVP, the higher the BNP values at baseline (Additional file 4), we found that disproportionately elevated BNP in relation to CVP after initial fluid resuscitation might predict increased mortality. Our findings may suggest that disastrous sepsis, except for increased BNP levels, is also associated with vascular underfilling, despite appropriate volume overexpansion [22], possibly due to extreme systemic vasoplegia and continuing fluid extravasation. It is intriguing to speculate that CVP-guided fluid resuscitation [22] might have been inadequate in some of our nonsurvivors; however, whether the BNP/CVP ratio may be of value in making decisions related to volume expansion by adding to established clinical data [22] should be elucidated in future studies.

In our septic patients, we found a greater incidence of RV than LV systolic dysfunction (Table 2). In addition, depressed RVEF, but not LVEF, showed an independent association with mortality (Table 4). However, RVEF and LVEF were strongly interrelated (Additional file 1), and both were significantly associated with serial BNP measurements (Table 3). Although mechanical ventilation and/or hypoxemia may increase PVRI, thus unmasking RV dysfunction, systemic vasoplegia may conceal impaired LV contractility, resulting in an artificially elevated LVEF [41]. We hypothesized that myocardial depression in sepsis is global rather than right-sided; however, our findings suggest that a PAC-derived RVEF may reflect septic cardiomyopathy better than echocardiography and may have greater diagnostic and prognostic value in mechanically ventilated septic patients.

We acknowledge that there are some points that have to be considered in the interpretation of our results. First, 28-day mortality was 56.7% in septic shock patients and 47.6% in critical sepsis patients. These mortality rates may be comparable to those reported by previous researchers who studied similar ICU patient populations [42,43], but they are higher than currently expected in the general population of critical sepsis patients [44]. Despite the fact that direct comparisons with historical studies are difficult because of differences in study populations and designs, these differences are likely to be due to the severity of critical illness and potential comorbidities. Second, LVEF values may have been affected by changes in cardiac loading conditions. Certainly, less load-dependent echocardiographic indices of LV contractility, such as tissue Doppler imaging–derived mitral annular velocities [45], might have provided further insight into the role of septic cardiomyopathy in BNP elevation. However, PAC-derived LVSWI, which is considered a less load-dependent index of LV contractility [46], also failed to demonstrate an independent association with BNP, probably suggesting that septic cardiomyopathy is not the major determinant of BNP rise. Third, dynamic indices of volume responsiveness (pulse pressure variation and stroke volume variation) might have identified responders to volume expansion more precisely than cardiac filling pressures (CVP and PCWP) [47]. However, CVP-guided fluid resuscitation was the existing clinical practice at the time our study was conducted [22]. Fourth, troponin measurements were not performed systematically in our patients, which is a potential limitation of our study. Newer, highly sensitive assays that enable clinicians to identify previously undetected pathological troponin levels [48] may provide more information on the role of myocardial damage in BNP elevation in septic patients.

Our study has several strengths and originality. We evaluated cardiac function and hemodynamics as potential determinants of BNP secretion by using both right heart catheterization and echocardiography. Moreover, we used a PAC equipped with a rapid response thermistor for the assessment of RV systolic function, as echocardiographic estimation of RV systolic function in mechanically ventilated ICU patients is considered problematic in terms of feasibility and reproducibility [49]. In addition, by comparing septic patients with hemorrhagic shock patients, we provide evidence that noradrenalin dose may not be a stimulus of BNP secretion *per se*.

## Conclusions

Our data clearly show that the severity of illness, rather than sepsis-induced myocardial depression, is the main determinant of BNP increase in mechanically ventilated patients with critical sepsis. Increased LV filling pressures and volume overexpansion during the acute phase of critical sepsis were not associated with BNP elevation in our series. Our findings also suggest that increased baseline BNP values >800 pg/ml and BNP/CVP ratio >126 pg/mmHg^1^/ml on day 2 may be early predictors of adverse outcomes. In addition, prolonged BNP elevation and inability to reduce BNP below the critical threshold of 500 pg/ml may also imply increased mortality. In this respect, our study results suggest that both baseline BNP values and BNP trends should be carefully assessed in the acute phase of critical sepsis and possibly considered in the management of the disease.

## Key messages

•The severity of critical illness, rather than septic cardiomyopathy, is probably the main determinant of BNP rise in critical sepsis patients.

•In septic shock patients, the noradrenalin dose is an index of critical illness rather than a stimulus for BNP secretion *per se*.

•Daily BNP concentrations are poorly associated with outcomes; however, early BNP elevation may be of clinical value in predicting mortality.

•BNP kinetics demonstrate a significantly faster decline over time in survivors than in nonsurvivors, in critical sepsis patients overall as well as in the subset of septic shock patients.

•In the subset of septic shock patients, an increased BNP/CVP ratio after initial fluid resuscitation and persistent BNP elevation >500 pg/ml may imply increased mortality.

## Abbreviations

APACHE II: Acute Physiology and Chronic Health Evaluation II; AUC: Area under the curve; BNP: B-type natriuretic peptide, CI, Cardiac index; CNS: Central nervous system; CVP: Central venous pressure; LV: Left ventricular; LVEF: Left ventricular ejection fraction; LVSWI: Left Ventricular Stroke Work Index; mABP: Mean arterial blood pressure; mPAP: Mean pulmonary arterial pressure; PAC: Pulmonary artery catheter, PEEP, Positive end-expiratory pressure; PaO_2_/FiO_2_: Ratio of partial pressure of oxygen in blood to oxygen concentration during mechanical ventilation; PCWP: Pulmonary capillary wedge pressure; PVRI: Pulmonary Vascular Resistance Index; ROC: Receiver operating characteristic; RV: Right ventricular, RVEF, Right ventricular ejection fraction; SD: Standard deviation; SE: Standard error; SOFA: Sequential Organ Failure Assessment; SVRI: Systemic Vascular Resistance Index.

## Competing interests

The authors declare that they have no competing interests.

## Authors’ contributions

JP participated in the design of the study, performed offline echocardiographic analysis, participated in the interpretation and statistical analysis of the data and drafted the manuscript. DM participated in the design of the study, performed statistical analysis and revised the manuscript critically for important intellectual content. MM participated in the collection of clinical data, performed BNP measurements and drafted part of the manuscript. EP participated in the collection of clinical data, performed BNP measurements and revised the manuscript critically for important intellectual content. PZ performed PAC-related hemodynamic measurements and revised the manuscript critically for important intellectual content. EZ conceived of the study and participated in its design, performed echocardiography, participated in the interpretation of data and revised the manuscript critically for important intellectual content. All authors read and approved the final manuscript.

## Supplementary Material

Additional file 1 Details of the results and further discussion. Click here for file

Additional file 2 **Five-day BNP kinetics in critically septic patients (*N*** **= 42) stratified by peak noradrenaline support (upper left), APACHE II score (upper right), RVEF (lower left) and total maximum SOFA score (lower right).** Click here for file

Additional file 3 Comparison of the percentage daily changes (relative to baseline) in SOFA scores and BNP values during the initial 5 days. Click here for file

Additional file 4 **Clinical determinants of BNP in septic shock patients (*N*** **= 30).** Click here for file

Additional file 5 Comparison of the characteristics of the patients who survived and those who did not survive up to day 28. Click here for file

Additional file 6 **Kaplan-Meier 28-day survival analysis of overall patients with critical sepsis (*N*** **= 42) stratified according to BNP concentration, RVEF and peak noradrenaline dose on day 1.** Click here for file

Additional file 7 The diagnostic performance of daily BNP measurements in predicting 28-day mortality. Click here for file
