# Supplementary material for: New insights into the mechanisms involved in B-type natriuretic peptide elevation and its prognostic value in septic patients
Source: Crit Care. 2014 May 9;18(3):R94. doi: 10.1186/cc13864 (PMC4075117; doi:10.1186/cc13864)
Supplement: Additional file 1 — Details of the results and further discussion. [file cc13864-S1.doc]

**Additional file 1.doc**

#### New insights into the mechanisms involved in B-type natriuretic peptide elevation and its prognostic value in septic patients

#### John Papanikolaou, Demosthenes Makris, Maria Mpaka, Eleni Palli, Paris Zygoulis, Epaminondas Zakynthinos

#### Department of Critical Care, School of Medicine, University of Thessaly, University Hospital of Larissa, Larissa, Thessaly, Greece

**Additional results**

Of our 42 patients with severe sepsis/septic shock, 16 were diagnosed with pneumonia (community-acquired/ventilator-associated: 4/12), 14 with bacteremia/catheter-related sepsis, five with peritonitis, two with cholocystitis, one with pyelonephritis, one with cellulitis, whereas in three patients the underlying cause was not established. Eighty percent (80%, 24/30) of our patients with septic shock and 58.3% (7/12) with severe sepsis demonstrated microbiological documentation of the disease; 23 patients had positive cultures for gram negative and four for gram positive bacteria, three demonstrated polymicrobial infection and one patient fungemia.

Co-morbidities in our patients included diabetes in 21.4% (9/42), hepatic disease in 11.9% (5/42), chronic respiratory disease in 21.4% (9/42), cancer or hematologic malignancy in 14.3% (6/42), autoimmune disease in 14.3% (6/42), chronic glucocorticoid therapy in 19% (8/42).

None of our septic shock patients was receiving dobutamine at the time of recruitment (exclusion criterion); five patients required dobutamine at some point due to severely decreased cardiac output or persistent lactic acidosis.

1. ***The incidence of LV and RV septic cardiomyopathy***

Five out of 42 (11.9%) critically septic patients presented moderately or severely depressed LVEF (<50%) [1], whereas 33/42 (78.6%) manifested moderately to severely reduced RVEF (<40%) [2]. LVEF and RVEF were strongly interrelated (Pearson’s correlation *r*=0.659, P<0.001).

Several indices reflecting the severity of critical sepsis, such APACHE II and SOFA scores, as well as peak noradrenaline dose, were associated with RVEF (Pearson’s correlation *r*=-0.508;-0.418;-0.402, P=0.001;0.006;0.008, respectively); among them, peak noradrenaline dose tended to affect independently RVEF in a multivariate linear regression model (regression coefficient *b*, -0.334, P=0.08). LVEF was associated only with APACHE II score (*r*= -0.335, P=0.03).

Neither LVEF nor RVEF were correlated with its corresponding filling pressures PCWP (P=0.187) and CVP (P=0.287), respectively; in addition, there were no significant differences between PCWP or CVP values among the grades of LVEF [1] or RVEF [2] dysfunction (one way ANOVA, P≥0.075 and P≥0.519, respectively).

1. ***Clinical determinants of BNP in septic shock***

In the subset of septic shock (N=30), the univariate determinants of BNP values are illustrated in *Additional Table 1 (Additional file 4.xls).* In this subgroup, the critical illness severity and peak noradrenaline dose remained the major determinants of BNP levels. Thus, APACHE II score was independently associated with BNP on day2 (regression coefficient *b*, 0.289, P=0.005) and maximum SOFA score with BNP on days2,3,5 (regression coefficient *b*: 0.362; 0.431; 0.567, P: =0.011; =0.036; =0.026, respectively). In addition, peak noradrenaline dose on day1 was independently associated with BNP on day1 (regression coefficient *b*=0.579, P=0.001). Similarly to the whole spectrum of critical sepsis, LVEF and RVEF in septic shock were significant, yet non independent determinants of BNP concentrations, while LV filling pressures showed no correlation with its corresponding BNP values. Interestingly, CVP on day1 and CVP/PCWP ratio on day2 were independent determinants of their corresponding BNP concentrations (regression coefficient *b*=0.224, P=0.05; *b*=0.310, P=0.033, respectively).

1. ***The relationship between the evolution of organ dysfunction and BNP in critical sepsis***

In our 42 critically septic patients, we assessed the relationship between the evolution of organ dysfunction and BNP by comparing the % daily changes (relative to baseline) in SOFA scores (indicating the evolution of organ dysfunction) and BNP, using linear mixed model analysis (*Additional file 3.tif*). We found that there was no significant difference in the way the two variables changed during the 5-day study period (P=0.157). In particular, survivors (N=22) were both SOFA and BNP decliners (although BNP demonstrated a faster decline over time than SOFA, P=0.014), whereas non-survivors demonstrated a similar (P=0.947) slightly upward trend in SOFA and BNP alteration over time.

1. ***Determinants of mortality in critical sepsis***

The comparisons of patient characteristics according to 28-day survival are presented in Additional Table 2 (*Additional file 5.xls).*

*Additional file 6.tif* illustrates the Kaplan-Meier 28-day survival curves of the 42 patients with severe sepsis/septic shock, stratified according to BNP, RVEF [2] and peak noradrenaline dose on day1.

1. ***Diagnostic performance of BNP in predicting mortality in critical sepsis and septic shock***

The diagnostic performance of daily BNP levels in predicting mortality was assessed by ROC curve-analysis in critical sepsis overall, as well as in the subset of septic shock (Additional Table 3, *Additional file 7.xls*). In critical sepsis, BNP values were of limited diagnostic accuracy in predicting 28-day mortality. BNP on day1 >800pg/mL and BNP on day2 >840pg/mL fairly predicted mortality with a sensitivity%, specificity% and area under the curve [AUC(95%CI)] of 65,64,0.70(0.54-0.86),P=0.03 and 65,64,0.68(0.52-0.84),P=0.044, respectively. In the subgroup of septic shock, BNP showed no prognostic value in ROC analysis.

**Additional discussion**

In the present study, we examined thermodilution-derived RVEF as a potential determinant of plasma BNP levels in critical sepsis. Interestingly, several lines of evidence point to a more important role of RV than LV dysfunction as a potential risk factor in critical sepsis. First, RVEF was better associated than LVEF with the severity of critical illness and BNP levels. Second, in septic shock, right ventricular filling pressures exerted an independent effect on BNP concentrations during the first two days, while LV filling pressures did not correlate with its corresponding BNP values. Finally, in contrast to LVEF, depressed RVEF showed an independent association with mortality in overall critical septic patients as well as in the subset of patients with septic shock (Table 4, main manuscript). Although RV dysfunction might be merely an epiphenomenon of intense illness, one may argue that early detection of depressed RV function and application of strategies that improve RV performance might be of major importance in critical sepsis.

**Additional figure legends**

***Additional file 2.tif: Five-day BNP kinetics in critically septic patients (N=42) stratified by peak noradrenaline support (upper left), APACHE II (upper right), RVEF (lower left) and total maximum SOFA score (lower right).*** Circles and vertical lines indicate mean BNP values and standard errors, respectively. The relative mean regression lines were constructed by using linear mixed model repeated measures. *P<0.05, Bonferroni’s subgroup analysis between lines’ mean intercepts (black hooks) and lines’ mean slopes (gray hooks). BNP= B-type natriuretic peptide; Noradrenaline= peak noradrenaline dose on day1; SE= standard error; MI= mean intercept; MS= mean slope; APACHE II=Acute Physiology and Chronic Health Evaluation Score II; RVEF=right ventricular ejection fraction; SOFA=total maximum Sequential Organ Failure Assessment.

***Additional file 3.tif: Comparison of the % daily changes in SOFA scores and BNP values during the initial five days.*** Δ%= % daily changes relative to baseline, SE= standard error; BNP= B-type natriuretic peptide; SOFA= Sequential Organ Failure Assessment.

***Additional file 6.tif: Kaplan-Meier 28-day survival analysis in overall patients with critical sepsis (N=42), stratified according to BNP, RVEF and peak noradrenaline dose on day1.*** The displayed P*-*values represent significant differences in 28-day survival in groups with BNP<524μg/mL, RVEF<30% and peak noradrenaline infusion≥17μg/min, by Log-rank (Mantel-Cox) test. BNP=B-type natriuretic peptide; RVEF=right ventricular ejection fraction.

**Additional tables**

***Additional Table 1 (Additional file 4.xls). Clinical determinants of BNP in septic shock (N=30).*** Clinical parameters associated significantly with serial BNP measurements on univariate analysis and independent determinants of BNP on the corresponding multivariate regression models.

|  | **Clinical determinants of BNP** | **Univariate linear regression analysis** | | |  | **Multivariate linear regression analysis** | | |
| --- | --- | --- | --- | --- | --- | --- | --- | --- |
|  |  | ***r*** | **R^2^** | **P** |  | **B (95%CI)** | ***b*** | **P** |
| **BNP (day 1)** | SOFA | 0.692 | 0.480 | <0.001 |  | 58.8 (-6 - 124) | 0.254 | 0.075 |
|  | Noradrenaline dose | 0.814 | 0.663 | <0.001 |  | 27.7 (13 - 124) | 0.579 | 0.001 |
|  | CVP | 0.437 | 0.191 | 0.016 |  | 42.2 (0 - 124) | 0.224 | 0.05 |
|  | LVEF | -0.491 | 0.241 | 0.006 |  | 2.4 (-14 - 124) | 0.054 | 0.76 |
|  | RVEF | -0.471 | 0.222 | 0.009 |  | -6.2 (-36 - 124) | -0.073 | 0.665 |
| **BNP (day 2)** | APACHE II | 0.386 | 0.149 | 0.035 |  | 50.9 (0 - 102) | 0.289 | 0.05 |
|  | SOFA | 0.651 | 0.424 | <0.001 |  | 113.3 (29 - 198) | 0.362 | 0.011 |
|  | CVP/PCWP | 0.425 | 0.181 | 0.024 |  | 1956 (0 - 3735) | 0.310 | 0.033 |
|  | LVEF | -0.578 | 0.334 | 0.001 |  | -20.5 (-47 – 5.9) | -0.325 | 0.122 |
|  | RVEF | -0.462 | 0.214 | 0.01 |  | 4.6 (-38 - 47) | 0.039 | 0.825 |
| **BNP (day 3)** | APACHE II | 0.521 | 0.271 | 0.005 |  | 35.2 (-12 - 83) | 0.224 | 0.138 |
|  | SOFA | 0.746 | 0.556 | <0.001 |  | 122 (9 - 235) | 0.431 | 0.036 |
|  | Noradrenaline dose | 0.680 | 0.462 | <0.001 |  | 12.8 (-13 - 39) | 0.199 | 0.316 |
|  | LVEF | -0.596 | 0.355 | 0.001 |  | -7.8 (-33 – 17) | -0.141 | 0.527 |
|  | RVEF | -0.522 | 0.272 | 0.005 |  | -4.9 (-51 - 41) | -0.045 | 0.825 |
| **BNP (day 4)** | APACHE II | 0.629 | 0.396 | 0.002 |  | 50.2 (-12 - 113) | 0.297 | 0.109 |
|  | SOFA | 0.750 | 0.563 | <0.001 |  | 97.3 (-27 - 221) | 0.363 | 0.117 |
|  | Noradrenaline dose | 0.763 | 0.582 | <0.001 |  | 19.8 (-13 - 53) | 0.292 | 0.221 |
|  | LVEF | -0.542 | 0.294 | 0.009 |  | 6.6 (-22 – 35) | 0.115 | 0.631 |
|  | RVEF | -0.510 | 0.260 | 0.015 |  | -22.4 (-73 - 28) | -0.195 | 0.362 |
| **BNP (day 5)** | APACHE II | 0.598 | 0.358 | 0.007 |  | 56.4(-5.6 - 118) | 0.337 | 0.071 |
|  | SOFA | 0.760 | 0.557 | <0.001 |  | 133.8(19 - 249) | 0.567 | 0.026 |
|  | Noradrenaline dose | 0.669 | 0.448 | 0.002 |  | -4(-41 - 33) | -0.060 | 0.817 |
|  | LVEF | -0.670 | 0.449 | 0.002 |  | -1.42(-30 - 28) | -0.028 | 0.917 |
|  | RVEF | -0.580 | 0.336 | 0.009 |  | -23.4(-68 - 21) | -0.237 | 0.278 |
| BNP= B-type natriuretic peptide;  *r*=Pearson’s correlation coefficient; R^2^= coefficient of determination; B,*b*= unstandardized and standardized beta coefficients; CI= confidence interval; APACHE II=Acute Physiology and Chronic Health Evaluation Score II; SOFA score=Sequential Organ Failure Assessment score; Noradrenaline dose= peak noradrenaline dose on day1; CVP= central venous pressure; PCWP= pulmonary capillary wedge pressure; LVEF= left ventricular ejection fraction; RVEF= right ventricular ejection fraction. | | | | | | | | |

***Additional Table 2 (Additional file 5.xls).* *Comparison of the characteristics of the patients having survived and those not having survived up to day 28.***

| **Characteristics** | **28-days survivors**  **(N=22)** | **28-day non-survivors**  **(N=20)** | **P** |
| --- | --- | --- | --- |
| Age | 57.95 (2.13) | 62.8 (2.17) | 0.12 |
| Sex [male(%)/female(%)] | 15 (68.2)/7(31.8) | 11 (55)/9(45) | 0.380 |
| Admitting diagnosis |  |  |  |
| Medical critical state | 14 (63.6) | 15 (75) |  |
| Surgical critical state | 5 (22.7) | 5 (25) |  |
| Multiple trauma | 3 (13.6) | 0 (0) |  |
| APACHE II, day1 | 16.5 (0.83) | 20.55 (0.94) | **0.002** |
| Peak noradrenaline dose, μg/min (day1) | 9.18 (2.26) | 19.5 (3.15) | **0.01** |
| Fluid balance, mL(day1) | 4697 (413) | 4473 (260) | 0.656 |
| Renal failure (day1) | 6 (27) | 5 (25) | 0.867 |
| Total maximum SOFA score | 9.32 (0.54) | 11.25 (0.47) | **0.011** |
| PO2/FIO2 | 338.9 (15.7) | 365.9 (14.7) | 0.22 |
| pH | 7.403(0.008) | 7.397 (0.01) | 0.675 |
| PEEP, mmHg | 5.95 (0.14) | 5.9 (0.18) | 0.808 |
| CVP, mmHg (day1) | 10.18 (0.42) | 9.45 (0.71) | 0.371 |
| CVP, mmHg (day2) | 9.86 (0.37) | 9.72 (0.52) | 0.822 |
| CVP, mmHg (day3) | 10.74 (0.49) | 10.87 (0.82) | 0.888 |
| PCWP, mmHg (day1) | 12.14 (0.64) | 12.75 (0.76) | 0.539 |
| PCWP, mmHg (day2) | 13.41 (0.54) | 14.30 (0.61) | 0.280 |
| PCWP, mmHg (day3) | 13.69 (0.51) | 13.69 (0.56) | 0.994 |
| mABP, mmHg | 72.5 (1.8) | 68.95 (2) | 0.193 |
| mPAP, mmHg | 24 (0.77) | 24.05 ( 0.9) | 0.966 |
| PVRI, dyn.sec.cm^-5^.m^2^ | 226.9 (23.2) | 193.7 (15.3) | 0.249 |
| SVRI, dyn.sec.cm^-5^.m^2^ | 1173.9 (54.6) | 1035.9 (47.2) | 0.065 |
| CI, L/min/m^2^ | 4.39 (0.19) | 4.68 (0.14) | 0.243 |
| SVI , mL/m^2^ | 41.1 (2.1) | 40.1 (1.5) | 0.697 |
| LVSWI, g.m.m^−2^ | 33.86 (2.1) | 30.65 (1.5) | 0.235 |
| RVEF, % | 36.59 (1.19) | 30.75 (1.17) | **0.001** |
| LVEF, % | 64.36 (2.88) | 61.15 (1.99) | 0.373 |
| BNP, pg/mL (day1) | 732.4 (122.5) | 1099.5 (133.8) | **0.049** |
| BNP, pg/mL (day2) | 843.7 (188.6) | 1113.2 (143.1) | 0.269 |
| BNP, pg/mL (day3) | 708.6 (151.1) | 975.8 (161.2) | 0.238 |
| BNP, pg/mL (day4) | 578.4 (131.9) | 960.5 (209.8) | 0.115 |
| BNP, pg/mL (day5) | 478.7 (119.9) | 816.7 (184.9) | 0.138 |
| Continuous data are presented as means±SE, categorical data as n(%).  APACHE II= Acute Physiology and Chronic Health Evaluation Score II; Renal failure= creatinine level≥2mg/dL or requirement for continuous renal substitution therapy; SOFA score= Sequential Organ Failure Assessment score; PaO_2_= partial pressure of oxygen; F_I_O_2_= fraction of inspired oxygen; PEEP= positive end-expiratory pressure; CVP= central venous pressure; PCWP= pulmonary capillary wedge pressure; mABP= mean arterial blood pressure; mPAP= mean pulmonary arterial pressure; CI= cardiac index; SVRI= systemic vascular resistance index; PVRI= pulmonary vascular resistance index; LVSWI= left ventricular stroke work index; RVEF= right ventricular ejection fraction; LVEF= left ventricular ejection fraction; BNP=B-type natriuretic peptide. | | | |

***Additional Table 3 (Additional file 7.xls). Diagnostic performance of daily BNP measurements in predicting 28-day mortality in critical sepsis and in septic shock.***

|  | **BNP cutoff, pg/mL** | **AUC± se** | **95% CI** | **Se (%)** | **Sp (%)** | **P value** |
| --- | --- | --- | --- | --- | --- | --- |
| **Critical sepsis overall (N=42)** | | | | | | |
| BNP on day1 | 800 | 0.7 ±0.08 | 0.54-0.86 | 65 | 64 | 0.030 |
| BNP on day2 | 840 | 0.68 ±0.08 | 0.52-0.84 | 65 | 64 | 0.044 |
| BNP on day3 | 835 | 0.65 ±0.09 | 0.48-0.83 | 65 | 64 | 0.106 |
| BNP on day4 | 445 | 0.67 ±0.10 | 0.46-0.87 | 75 | 64 | 0.109 |
| BNP on day5 | 501 | 0.68 ±0.13 | 0.43-0.93 | 67 | 82 | 0.117 |
| **Septic shock (N=30)** | | | | | | |
| BNP on day1 | 1128 | 0.6 ±0.11 | 0.39-0.81 | 53 | 69 | 0.357 |
| BNP on day2 | 1215 | 0.61 ±0.11 | 0.4-0.82 | 59 | 69 | 0.325 |
| BNP on day3 | 937 | 0.58 ±0.11 | 0.36-0.81 | 57 | 61 | 0.467 |
| BNP on day4 | 1145 | 0.62 ±0.14 | 0.34-0.89 | 56 | 92 | 0.367 |
| BNP on day5 | 1230 | 0.69 ±0.16 | 0.37-1 | 67 | 92 | 0.203 |
| BNP=B-type natriuretic peptide; auc= Under the Curve; SE= Standard Error; CI= confidence interval; Se (%)= sensitivity (%); Sp (%)= specificity (%) | | | | | | |

**Additional references**

1. [Brueckmann M](http://www.ncbi.nlm.nih.gov/pubmed?term=Brueckmann M%5BAuthor%5D&cauthor=true&cauthor_uid=16027260), [Huhle G](http://www.ncbi.nlm.nih.gov/pubmed?term=Huhle G%5BAuthor%5D&cauthor=true&cauthor_uid=16027260), [Lang S](http://www.ncbi.nlm.nih.gov/pubmed?term=Lang S%5BAuthor%5D&cauthor=true&cauthor_uid=16027260), et al: **Prognostic value of plasma N-terminal pro-brain natriuretic peptide in patients with severe sepsis.** *[Circulation.](http://www.ncbi.nlm.nih.gov/pubmed/16027260#%23) 2005,* **112**:527-534.
2. [Schulman DS](http://www.ncbi.nlm.nih.gov/pubmed?term=Schulman DS%5BAuthor%5D&cauthor=true&cauthor_uid=3276186), [Biondi JW](http://www.ncbi.nlm.nih.gov/pubmed?term=Biondi JW%5BAuthor%5D&cauthor=true&cauthor_uid=3276186), [Matthay RA](http://www.ncbi.nlm.nih.gov/pubmed?term=Matthay RA%5BAuthor%5D&cauthor=true&cauthor_uid=3276186), [Barash PG](http://www.ncbi.nlm.nih.gov/pubmed?term=Barash PG%5BAuthor%5D&cauthor=true&cauthor_uid=3276186), [Zaret BL](http://www.ncbi.nlm.nih.gov/pubmed?term=Zaret BL%5BAuthor%5D&cauthor=true&cauthor_uid=3276186), [Soufer R](http://www.ncbi.nlm.nih.gov/pubmed?term=Soufer R%5BAuthor%5D&cauthor=true&cauthor_uid=3276186): **Effect of positive end-expiratory pressure on right ventricular performance. Importance of baseline right ventricular function.** *[Am J Med.](http://www.ncbi.nlm.nih.gov/pubmed/3276186#%23) 1988*, **84**:57-67.
