# Supplementary figures and images for: New insights into the mechanisms involved in B-type natriuretic peptide elevation and its prognostic value in septic patients
Source: Crit Care. 2014 May 9;18(3):R94. doi: 10.1186/cc13864 (PMC4075117; doi:10.1186/cc13864)

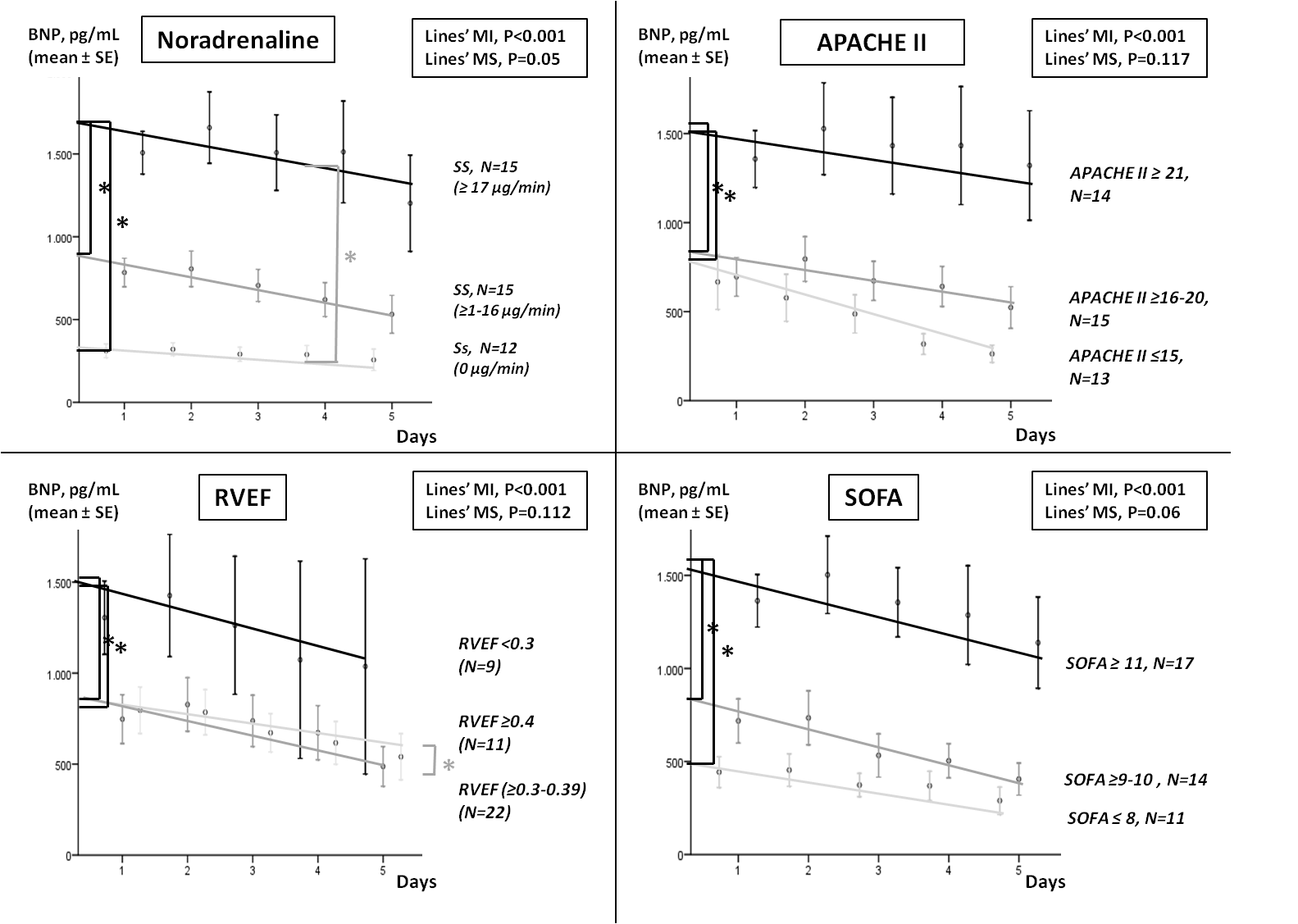

Supplement: Additional file 2 — Five-day BNP kinetics in critically septic patients (N = 42) stratified by peak noradrenaline support (upper left), APACHE II score (upper right), RVEF (lower left) and total maximum SOFA score (lower right). [file cc13864-S2.tiff]

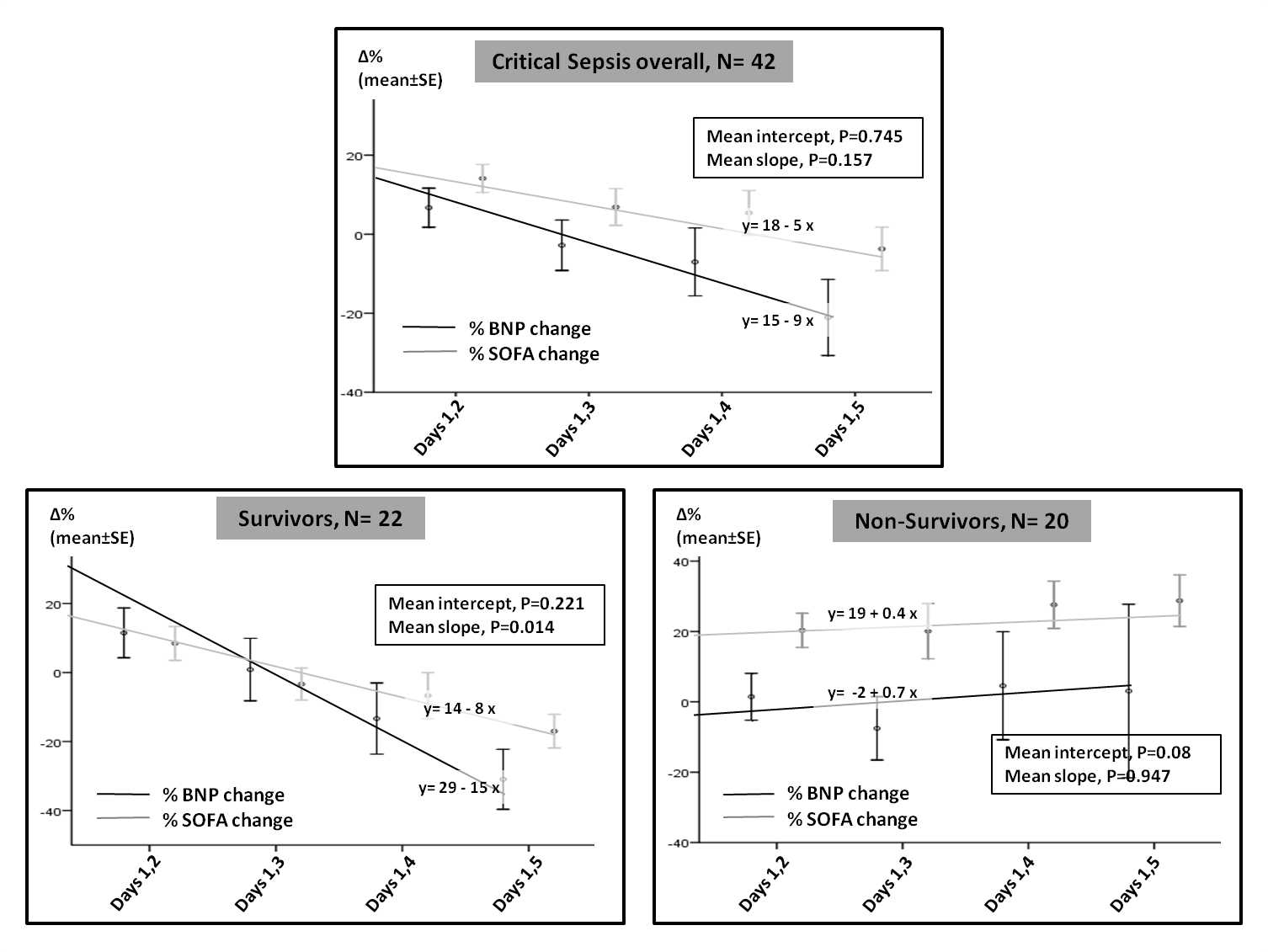

Supplement: Additional file 3 — Comparison of the percentage daily changes (relative to baseline) in SOFA scores and BNP values during the initial 5 days. [file cc13864-S3.tiff]

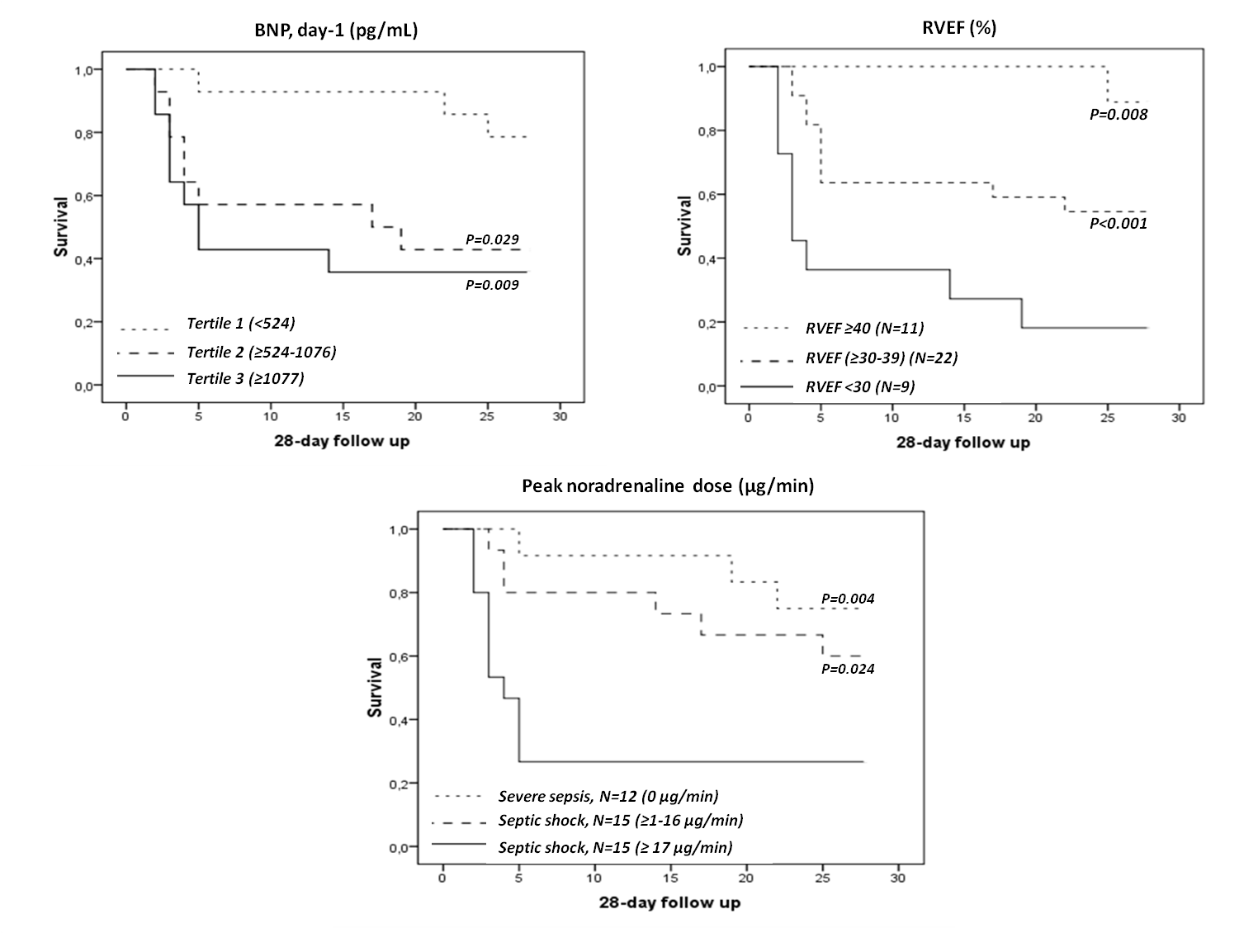

Supplement: Additional file 6 — Kaplan-Meier 28-day survival analysis of overall patients with critical sepsis (N = 42) stratified according to BNP concentration, RVEF and peak noradrenaline dose on day 1. [file cc13864-S6.tiff]
